# Consequence of intraventricular hemorrhage on neurovascular coupling evoked by speech syllables in preterm neonates

**DOI:** 10.1016/j.dcn.2018.01.001

**Published:** 2018-01-05

**Authors:** Mahdi Mahmoudzadeh, Ghislaine Dehaene-Lambertz, Guy Kongolo, Marc Fournier, Sabrina Goudjil, Fabrice Wallois

**Affiliations:** a INSERM U1105, Université de Picardie, CURS, Amiens, France; b INSERM U1105, Unit Exploration Fonctionnelles du Système Nerveux Pédiatrique, South University Hospital, Amiens, France; c Cognitive Neuroimaging Unit, CEA DSV/I2BM, INSERM, CNRS, Université Paris-Sud, Université Paris-Saclay, NeuroSpin Center, 91191 Gif/Yvette, France; d INSERM U1105, Neonatal ICU, South University Hospital, Amiens, France

**Keywords:** Electroencephalogram (EEG), Functional Near Infrared Spectroscopy (fNIRS), Intraventricular hemorrhage, Neurovascular coupling, Preterm

## Abstract

Intraventricular Hemorrhage (IVH) is the leading cause of neurological and cognitive impairment in preterm neonates with an incidence that increases with increasing prematurity. In the present study, we tested how preterm neonates with IVH react to external stimulation (i.e. speech syllables). We compared their neural responses measured by electroencephalography (EEG), and hemodynamic responses measured by functional near-infrared spectroscopy (fNIRS), with those of healthy preterms. A neural response to syllables was observed in these infants, but did not induce a vascular response in contrast with healthy neonates. These results clearly demonstrate that the cerebral vascular network in IVH preterm neonates was unable to compensate for the increased metabolism resulting from neuronal activation in response to external stimulation. Optical imaging is thus a sensitive tool to identify altered cerebral hemodynamic in critically ill preterms before behavioral changes are manifested or when only minor abnormalities on other functional monitoring techniques such as EEG are visible. We propose that a multi-modal approach provides unique opportunities for early monitoring of cognitive functions and opens up new possibilities for clinical care and recommended practices by studying the difficulties of the premature brain to adapt to its environment.

## Highlights

• Cerebral hemodynamic response is unable to adapt to exogenous stimulation in IVH preterms.
• EEG–NIRS coregistration allows to obtain high temporal and spatial resolution measurements in preterm neonates.
• Optical imaging identifies pathological cerebral hemodynamics.

## 1 Introduction

Efficient brain functions depend on the integrity of the neural system that processes and produces information and the vascular system that provides oxygen and other energetic substrates. They also rely on the functionality of the fine tuning between the two systems as defined by the neurovascular coupling. Intraventricular hemorrhage (IVH) is the most common neurovascular complication of prematurity. It can be considered as a model of an alteration of the vascular system at this early period of development. It occurs very early, during the first 3 days of life. The etiopathogenesis of IVH is complex, but mainly involves fragility of the capillaries in the germinal matrix (GM), which are sensitive to anoxic mechanisms. IVH is graded from I to IV, according to the degree of bleeding and extension from the germinal matrix in which the first bleeding into the cerebral parenchyma occurs. Accumulation of blood inside the ventricles (grade I and II) can lead to ventricular distension (grade III) and rupture of the ventricular walls, resulting in extension of bleeding inside the parenchyma (grade IV) (Larroque et al., 2003). Infants with IVH have a lower and poorly regulated cerebral blood flow (Alderliesten et al., 2013; Perlman et al., 1983; Van Bel et al., 1987).

Although hemodynamic responses evolve in the course of development (Colonnese et al., 2008; Nourhashemi et al., 2017), a neurovascular coupling (NVC) can be observed with Near Infrared Spectroscopy (NIRS) in early premature neonates in different physiological conditions including spontaneous bursts of ongoing cortical activity (Roche-Labarbe et al., 2007) and in response to external stimulation in different domains: nociception (Bartocci et al., 2006; Maimon et al., 2013), somatosensory (Bartocci et al., 2006), visual (Colonnese et al., 2010) and auditory perception (Bisiacchi et al., 2009). It is also seen in epileptic discharges in premature (Wallois et al., 2010). As intraventricular Haemorrhage (IVH) affects the neurovascular coupling to spontaneous bursts of cortical activities (Roche-Labarbe et al., 2007), it might also impact the vascular supply following external stimulation and may be part of the negative consequences of IVH on cognitive development. Impairments of higher cognitive functions, including language (Magill-Evans et al., 2002), are common in IVH premature. Therefore it is mandatory to develop new approach to address the efficiency of the neuro-vascular interactions at this early stage of development considering the strong cognitive impact that might have such a precocious dysfunction.

In our previous work using both High Density EEG (HD-EEG) and High Density functional Near Infrared Spectroscopy (HD-fNIRS), we have shown that 28–30 wGA healthy neonates already process several dimensions of speech. They react to a change of phoneme (ba vs. ga) and to a change of voice (male vs. female) by an increase in the neurovascular coupling, using parallel networks within the superior temporal regions and extending into the inferior frontal regions (Mahmoudzadeh et al., 2013; Mahmoudzadeh et al., 2017) with a very similar pattern to that observed in full-term neonates (Pena et al., 2003; Telkemeyer et al., 2009), older infants (Hyde et al., 2010) and adults (Celsis et al., 1999). We therefore used our experimental paradigm in a small group of eight 28–32 wGA neonates with high-grade IVH to examine whether severely injured premature vascular networks are able to react to exogenous quas i ecologic stimulation and how the two arms of the neurovascular system adapt to stimulation when the vascular system is altered. We recorded the neural response by high-density EEG and the vascular response by optical imaging while these infants were listening to speech syllables. We compared the results to previously published results obtained in age-matched healthy preterms (Mahmoudzadeh et al., 2013; Mahmoudzadeh et al., 2017). We hypothesized that during the risk period for IVH, immature germinal matrix which is richly supplied with microvessels lack tight neurovascular junctions. Thus, cerebral hemodynamic response might be unable to adapt to exogenous stimulation in infants with IVH.

## 2 Materials and methods

### 2.1 Participants

Eight preterm neonates (6 males; mean gestational age (GA) at test: 30.5 weeks GA, Table 1) with high-grade (III or IV) intraventricular hemorrhage were tested at a median postnatal age of 7 days (range 1–36) and compared to previously published data obtained in 12 healthy preterm neonates by optical topography (8 Males, mean GA at test: 30.7 weeks GA ± 1.5) and in 19 healthy preterm neonates by Event–Related–Potentials (ERPs) (11 males; mean GA at test: 30.4 weeks GA ± 1.4) (Mahmoudzadeh et al., 2013; Mahmoudzadeh et al., 2017). The patients were recruited as IVH grade III and IV based on the grading of severity of Germinal Matrix–Intraventricular Hemorrhage (GM-IVH) by Ultrasound Scan. MRI was not preformed because of ethical issue due to the challenge to move clinically instable neonates to the MRI for pure research reasons as the MRI is not in the vicinity of the neonatal unit. While MRI has the advantage of superbly displaying soft tissue contrast differentiation and the exact extent and site of brain injury, transcranial ultrasonography can be performed at the bedside, and is a relative sensitive brain imaging method to assess lesions and tissue vascularisation. (Lagercrantz et al., 2010). As it is the common case, clinical EEG of the preterms with IVH was abnormal (except one neonate, see Table 1). The altered background activities associated with intraventricular hemorrhages have been classically demonstrated (i.e. occurrence of Positive Rolandic Sharp Waves, or Positive Temporal Spike and also the normal “tracé discontinu” background pattern is sometimes replaced by burst suppression).

**Table 1:** Clinical features of the IVH infants tested.

Infant No.	Gender	GA at birth (weeks)	GA at test (weeks)	Birth Weight (g)	EEG	Apgar 1 min	Apgar 5 min	Brain US	Delivery	Presentation	Clinical conditions (Etiology)
1	M	29 4/7	30	1330	A	6	6	IVH3	Cesarean	–	Twin
2	M	25 4/7	28 5/7	950	A	3	5	IVH3	Vaginal	Cephalic	metrorrhagia
3	M	27 2/7	32	1160	A	8	8	IVH3	Cesarean	–	PROM, chorioamnionitis
4	F	30 3/7	31 6/7	1450	N	9	10	IVH3 Bilateral	Cesarean	transverse	placenta praevia, AFH
5	M	31 1/7	31 4/7	1080	A	10	10	IVH4	Cesarean	Cephalic	Twin
6	F	27	32 1/7	995	A	0	5	IVH4	Vaginal	Breech	Twin, Preeclampsia
7	M	26 5/7	29 2/7	1000	A	3	6	IVH4/3	Vaginal	–	Twin
8*	M	28 3/7	29	1200	A	8	9	IVH3	Vaginal	Cephalic	Twin

M: Male, F: Female, GA: Gestational Age, EEG: ElectroEncephaloGram, Cranial US: Cranial ultrasound, N: Normal, A: Abnormal, RPH: RetroPlacental Hematoma, PROM: Premature Rupture Of Membranes. AFH: Acute fetal hypoxia (* subject was removed from optical imaging study).

ERP and optical topography were obtained successively in each IVH infant in random order. The data for one IVH preterm were discarded, as no useful functional data were obtained due to poor positioning of the optical probe. The parents were informed on the goals of the study and provided their written informed consent. The study was approved by the local ethics committee of Amiens University Hospital (CPP Nord-Ouest II) according to the guidelines of the Declaration of Helsinki of 1975 (ref ID-RCB 2008-A00728-47).

### 2.2 Experimental paradigm

**Fig. 1:**
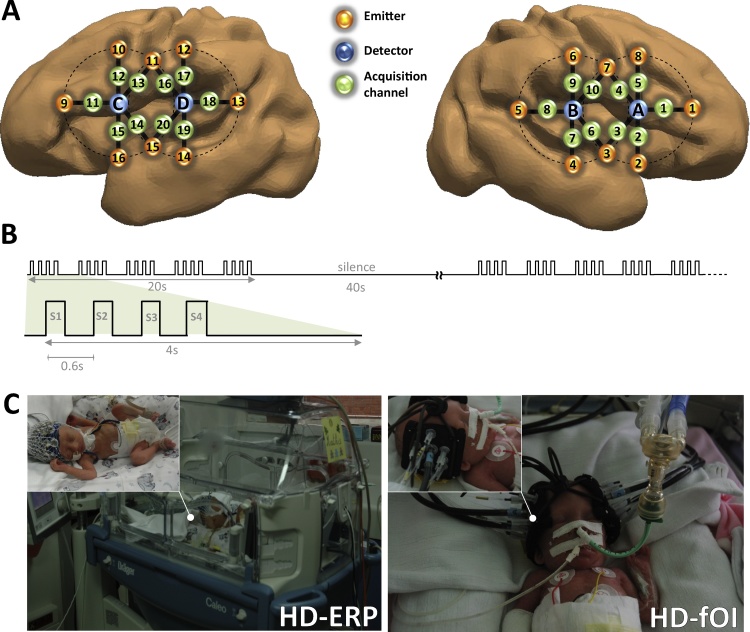
(A) Estimated projection of the optodes on the brain of a 30 w GA preterm infant. The geometric layout of the optical probe and its projection on the brain mesh of an individual 30 wGA preterm infant (courtesy of P. Hüppi and J. Dubois). Numbers 1–16 correspond to the light sources and letters A, B, C and D correspond to the detectors. (B) Auditory stimulation paradigm. Five series of four syllables were presented (SOA = 600 ms) in a block (duration 20 s), followed by 40 s of silence. Three types of blocks were randomly presented for a total duration of 108 min. In standard blocks, the same syllable (/ba/or/ga/) was presented, whereas, in deviant blocks, a change of voice (DV) or a change of phoneme (DP) was presented three times. (C) HB-ERP and HD-fOI system. Left: Preterm neonate with high-density EEG cap. The infants were tested while sleeping at night to avoid daylight and the intense daytime activity of a neonatal care unit. They were placed in the supine position on a comfortable pad in a dark and quiet incubator. The incubator was further protected from ambient light by dark sheets. Stimuli were binaurally presented at a comfortable hearing level (≈70 dB) via speakers placed at the infant’s feet, at a distance of 30 cm from the subject’s head. Right: A special probe made from soft, flexible foam (thickness = 5 mm) was designed to comfortably maintain the source and detector fibers on the infant’s head. A patch comprising two detectors and eight emitters (optodes) perpendicular to the head surface was placed on each side of the head. Each of the eight optodes contained two wavelength emitter glass fibers (690 and 830 nm). The probe was smoothly secured to the infant’s head to cover the perisylvian areas on each side with straps and foam padding. The probe was designed as a round-grid layout, maintaining a distance of 15 mm between the optodes (Fig. 1A). This layout allowed 10 measuring points (channels) that were simultaneously sampled on each hemisphere.

Four syllables (/ba/and/ga/, produced by male and female speakers) were matched for intonation, intensity, total duration (285 ms), prevoicing and voiced formant transition duration (40/45 ms) (Mahmoudzadeh et al., 2013; Mahmoudzadeh et al., 2017). They were presented at a comfortable hearing level (≈70 dB) via speakers placed at the infant’s feet, by series of four (Stimulus Onset Asynchrony = 600 ms) according to a block design (20 s of stimulation followed by 40 s of silence) for a total duration of 108 min. Each block comprised 20 syllables (five 4-syllable trials separated by an inter-trial interval of 1600 ms (Fig. 1B)). The design comprised three types of blocks in which the responses to a change of voice (male *vs.* female) and a change of phoneme (ba *vs*. ga) could have been analyzed. However, given the weak response in each individual and the small number of infants, we focused on the main response to syllables and we merged all blocks and all types of trials as a lack of a significant response to a change of syllable in these conditions can be related to a weak statistical power rather than to a genuine deficit. The sleep state was checked during EEG analysis based on the EEG cardiac and respiratory features by the experienced clinical neurophysiologist (F.W.) (Wallois, 2010).

### 2.3 HD-ERP recording

EEG was recorded at the bedside using Ag/AgCl surface electrodes and a nasion reference at a sampling rate of 2048 Hz, amplified by A.N.T^®^ (Enschede, The Netherlands) and DC–50 Hz filtered. The impedance of electrodes was kept below 5 kΩ. The number of electrodes (31–61) was determined by the infant’s head circumference in order to maintain a regular inter-electrode space (center-to-center) of about 1.5 cm. Four caps were used to cover the range of head circumferences observed at this age. In all infants, a minimum of 31 electrodes were placed on the classical 10–20 points and supplementary electrodes were placed on intermediate positions according to head circumference.

### 2.4 HD-fOI recording

We used a multichannel frequency-domain-based optical imaging system (Imagent, ISS Inc., Champaign, IL) to acquire changes in oxygenated hemoglobin and deoxygenated hemoglobin concentrations during the auditory stimulation task. Imagent is a frequency-domain tissue spectrometer comprising 32 intensity-modulated laser diodes at two wavelengths (λ = 690 nm and 830 nm) coupled to optical fibers, and four gain-modulated photomultiplier tube (PMT) detectors to collect the signal separately at both wavelengths. The modulation frequency of the laser intensity was 110 MHz, and the cross-correlation frequency for heterodyne detection was 5 kHz. The reflected light was collected in photomultiplier tubes (PMT) and demodulated. The average output power of the lasers was about 0.5 mW and the acquisition rate of the optical system was 9.1912 Hz (about one sample every 110 ms). To evaluate the sleep state during HD-NIRS recording, the changes in respiration and any ocular movement were precisely noted by the well experienced clinical electrophysiologist technician, based on the classical criteria (Lamblin et al., 1999).

#### 2.4.1 Optical imaging probe

A special probe made from soft, flexible foam (thickness = 5 mm) was designed to comfortably maintain the source and detector fibers on the infant’s head. A patch comprising two detectors and eight emitters (optodes) (resulting in 20 channels) perpendicular to the head surface was placed on each side of the head. Each of the eight optodes contained two wavelength emitter glass fibers (690 and 830 nm). Light in these two wavelengths is absorbed differently by oxyhemoglobin and deoxyhemoglobin, thereby allowing calculation of their respective concentrations in the medium. To define the best distance between emitters and detctors we considered that the distance between the temporal cortex and the scalp was about 7 mm. There is no reason to think that the scalp-brain distance would be different in IVH infants since IVH is a bleeding in the ventricles. We may even expect a decrease of the scalp-cortex distance because of the ventricular dilatation that often occurred and pushes the brain towards the skull. In any case, head size was similar in both groups confirming that there was no hydrocephaly or dilatation of the head as reported by ultrasound. We thus designed a round-grid layout, keeping a distance of 15 mm between the optodes (Fig. 1). This layout allowed 10 measuring points (hereafter called channels) that were simultaneously sampled on each hemisphere.

As the precedent study, our initial goal was to simultaneously record HD-NIRS and HR-ERPs. Therefore we designed an experimental protocol which fitted the constraints of both methods. Due to technical difficulties and lack of space in the design of a probe carrying both optodes and electrodes, we did not succeed to record HD-NIRS and HR-ERPs at the same time. To maximize reproducibility, we paid attention to conduct both studies on the same day and with the same design. It is unlikely that age or treatment would have been compelling parameters for the reproducibility of the effect of the task performed at few minutes or hours distance. Although ERPs were extracted from discontinuity periods during quite sleep while NIRS was continuously monitored over discontinuity periods and bursts of activity. Because the burst of activity induced a diffuse not localized neurovascular coupling (Roche-Labarbe et al., 2007) that is much smaller than the one evoked by exogenous stimuli, it might impact only slightly and non-selectively the exogenous localized evoked responses.

### 2.5 Data processing and statistical analyses

Data processing and statistical analysis were similar to those performed in our previous studies in healthy preterm neonates (Mahmoudzadeh et al., 2013; Mahmoudzadeh et al., 2017). StO2 was calculated as [HbO]/([HbO] + [Hb]) (for more details see (Hueber et al., 1999; Metz et al., 2013).

### 2.6 HD-fOI data processing

The Modified Beer Lambert Law was applied to the signals at the two wavelengths (690 and 830 nm) in order to convert the signal intensities into relative changes in (de)oxy-hemoglobin concentration. A z-score-based algorithm was used to reject artifacted signals: as individual features, such as skull thickness and hair color, influence signal strength. It was also employed to normalize the data between subjects as Z-score based algorithm converts all signals to a common scale with an average of zero and standard deviation of one. The block was marked for rejection if at any time during the 30 s of the time-window [−5 +25s], this value exceeded the threshold (z-score: 4). This procedure was applied to each channel. When one or more channels contained an artifact, the entire block was rejected. The remaining cleaned [HbO] and [Hb] signals were band-pass filtered [0.03–0.5 Hz] using a zero phase filter (Butterworth, order: 6) to eliminate physiologic noise (e.g. slow drifts, arterial pulse oscillations ∼164 BPM, Beats Per Minutes). Both signals were segmented relative to the onset of each block (−5 to +25 s). A linear detrend, followed by a baseline correction on the 5 s preceding the onset of the block were performed on the segments [−5, 25 s]. Finally, the segments were averaged in each subject for [Hb] and [HbO]. An average of 75 blocks were included for healthy infants (61–96 blocks), and 78 for IVH infants (67–99 blocks)

EEG at this age consists of bursts of high voltage activity followed by period of weak voltage (“Tracé discontinu”). These bursts of activity could affect the hemodynamic response but could not be discarded because the EEG and NIRS were not simultaneously recorded. However, assuming that spontaneous bursts of electrical activity are random relative to the stimulation, the hemodynamic response to this endogenous activity is neutralized through the average process, and thus should not affect the hemodynamic response locked to the exogenous stimuli. In addition, cerebral hemodynamic responses induced by endogenous activity in preterm neonates (both healthy and pathological cases) have a relatively poor signal-to-noise ratio (Roche-Labarbe et al., 2007) compared to hemodynamic evoked response due to exogenous stimuli (Fig. 3 current study).

### 2.7 Optical data visualization

In order to visualize the location of the brain activity measured with our optical system, we used a realistic head model of a 30 wGA premature neonate, provided by J. Dubois and P. Hüppi (Department of Pediatrics, Geneva University Hospitals, Geneva, Switzerland) (see (Dubois et al., 2008) for a precise description of the steps to obtain a head and brain mesh from T2w magnetic resonance images). A virtual layout was applied on the head model using the same preauricular biomarkers as reference positions as those of real infants. This layout was used to determine the position of each virtual NIRS acquisition channel relative to the preterm’s brain (Fig. 1). Each pair of NIRS emitters and detectors was then used to compute the optical signal propagation in the brain according to a photon migration model (Sassaroli et al., 2006). This step resulted in brain volume filling with information following the “banana shape” of the light propagation between emitters and each of the neighboring detectors, using the photon path modeled by the prescribed hitting density function. The individual photon migration probability distributions obtained were then combined using a tricubic spatial interpolation step in order to consider the interaction between the individual distributions. For visualization purposes, the volume-based information was projected onto the infant’s brain areas covered by the optical probe with a color scale proportional to the amplitude of the response.

The mean signal obtained in each subject was averaged over the 12/7 normal/IVH infants (grand average) for [Hb] and [HbO]. At each time step (every ∼110 ms) of the sampling frequency (9.19 Hz) of these grand averages, we computed a surface-based topographic color map as described above to create a video animation of the brain activity throughout the duration of stimulation (Video S1).

### 2.8 Statistical analysis

Hemodynamic response can be characterized by two parameters: amplitude and duration of the response, that are summarized by the area under the curve (AUC) computed for each channel and in each infant. The AUC provides information reflecting the accumulative variation of HbO concentration during the whole stimulation period. Therefore, AUC is more suitable than an amplitude peak difficult to evaluate during long event. T-test comparisons were performed between the two groups on the mean AUC over the left and right hemisphere. Auditory responses in our preterms were assessed by means of two parameters, amplitude and AUC of the response, according to the following strategy. The number of points for each analysis had to be decreased in order to resolve the problem of repeated measures (20 channels × 30 time bins = 600). An elegant solution proposed by Maris and Oostenveld (Maris and Oostenveld, 2007), initially for electrophysiological recordings with dense arrays of captors, is to use cluster-based statistics. Thus to control the risk of false-positive or familywise error, due to multiple comparisons, we used nonparametric statistical tests as implemented in Fieldtrip (Oostenveld et al., 2011), a toolbox developed in Matlab (available at http://fieldtrip.fcdonders.nl) by these authors. We downsampled the signal to 1 Hz in the studied time-window (−5 +25 s) to decrease the number of measurements. This downsampling does not affect the quality of the measurements in view of the slow hemodynamic response. We used cluster-based statistics on the 30 time bins × 20 channels to automatically identify, without prior hypotheses, spatial and temporal clusters showing a significant difference between healthy and IVH conditions (Fig. S1) (for more detail please see Mahmoudzadeh et al. (2013)). We limited our analyses to the HbO signal, which presented a better signal-to-noise ratio than Hb (Bortfeld et al., 2009; Bortfeld et al., 2007; Homae et al., 2006; Homae et al., 2007; Minagawa-Kawai et al., 2007; Pena et al., 2003; Saito et al., 2007).

### 2.9 HD-ERP data processing

**Fig. 2:**
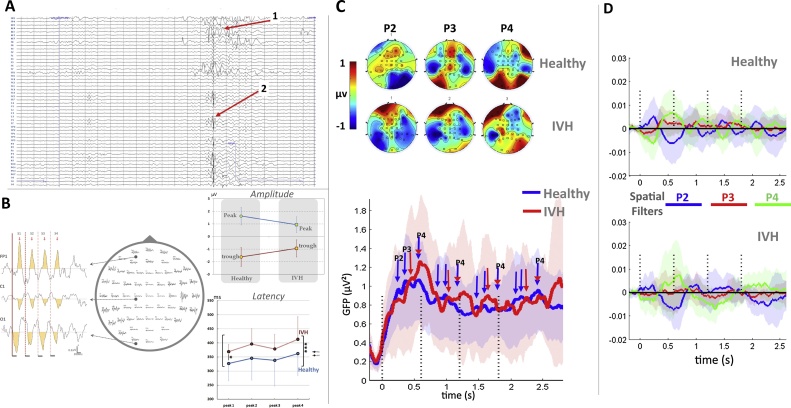
(A) High-resolution EEG. Recording of an individual IVH neonate showing pathological features (1) Abnormal frontal slow waves, (2) positive temporoparietal spikes and increased discontinuity. (B) Grand-average plot of the ERPs in IVH preterms; on the middle, response from each electrode and, on the left, three electrodes have been selected to show the decrease in amplitude with syllable repetition, especially observed between S1 and S2; on the right-up shows that IVH infants have smaller deflections than healthy infants, the right-down shows the difference in peak latencies (*** Three asterisks show significantly different peak latencies (pooled peaks S1-S4) between healthy and IVH infants (p < 0.001). * One asterisk indicates that the first peak latency (S1) is significantly (p < 0.05) different between healthy and IVH infants). (C) 2D maps of peaks 2, 3 and 4 in healthy and IVH preterm neonates. The maps are computed on a 40 ms time-window centered on 244 (P2), 378 (P3) and 576 ms (P4) after the first syllable of the trials. (D) Mean and standard deviation of the global field power (GFP) in healthy (blue) and IVH (red) neonates. The dotted lines indicate the onset of each of the 4 syllables of a trial. The amplitude of the response decreased with syllable repetition, but the peaks remained visible every 600 ms. Note the marked variability across subjects, especially in the IVH group due to the small number of subjects but also to the impact of the lesion. (E) Grand average (and standard deviation) of the projections of each pathological and healthy individual recording on spatial templates corresponding to the 2D maps of P2, P3 and P4 in healthy subjects. The repetition of the topographies after each syllable is revealed by the repetition of the same pattern of successive increase of the three coefficients after each syllable. Although the pattern is less obvious in IVH neonates, no significant difference was observed between the groups. (For interpretation of the references to colour in this figure legend, the reader is referred to the web version of this article.)

EEG was band-pass filtered (1–20 Hz), down-sampled to 512 Hz, segmented in 4 s epochs ([−0.5 +3.5]) time-locked to the first syllable of the trial, and baseline corrected to the 200 ms before S1 onset. At this age, background EEG is marked by long periods of low voltage activity (around 10 μV) interrupted by bursts of high voltage activity with an amplitude of about 300 μV. We used an automatic artifact-rejection procedure to discard these high-activity periods first because this high-voltage background activity would prevent detection of ERPs (i.e. the signal to noise ratio is too low) and second because neonates do not present the same neural state during these periods and the silent period. The thresholds for the automatic rejection procedure were determined after visual inspection of the data in order to reject bursts of high voltage activity: Individual channels were rejected when their absolute amplitude exceeded 50 μV or when a local amplitude jump between ten successive time-points exceeded 30 μV. A channel was rejected for the entire session if it was rejected on more than 70% of the trials. The entire trial was rejected if more than 15% of the channels were rejected. An average of 319 trials were included for healthy infants (141–510 trials), and 248 for IVH infants (77–493 trials). To obtain a smooth topography for all head sizes, we used interpolation for areas in between the available electrodes (Fig. 2c). The 2D spline interpolation accounts for the different geometry of the head using one value for each channel and the x and y coordinates, the values between electrodes are interpolated and plotted.

**Fig. 3:**
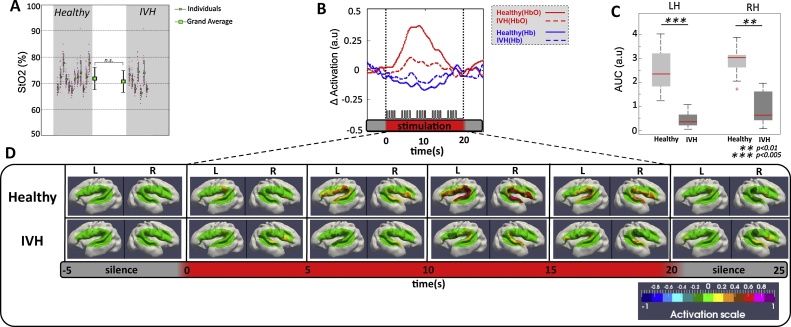
(A) Mean StO_2_ of Healthy (12 preterms) and IVH (7 preterms) infants during the experiment. Each thin black error bar indicates the mean and standard deviation across the 20 NIRS channels (red dots). Thick black error bars indicate mean and standard deviation of StO_2_ over Healthy and IVH preterm infants. (B) shows one sample fOI channel of grand-average of HbO (red lines), Hb (blue lines) changes for healthy (solid lines) and IVH (dotted lines) neonates during auditory stimulation. Horizontal red thick bars indicate the period of the stimulation (20 s). This response was pronounced for the healthy neonates, with a significant increase in HbO. (C) Comparison of the mean AUC changes of Oxy-Hb induced by auditory stimulation between healthy and IVH infants. For each hemisphere, activations were significantly reduced in the IVH group compared to the healthy group. (D) Topographies of the hemodynamic response in the two groups (Healthy, IVH). The HbO response, averaged each 5 s, was projected on a 3-D mesh of an individual 30 wGA preterm neonate. The red rectangle indicates the duration of auditory stimulation. The hemodynamic response in both hemispheres was weak in IVH neonates compared to healthy infants. (For interpretation of the references to colour in this figure legend, the reader is referred to the web version of this article.)

For statistical analysis, we considered the 43 most common electrodes among the IVH group (AFz, Fz, FCz, Cz, CPz, Pz, Oz on the midline and AF8, AF4, F8, F6, F4, F2, FC4, FC2, C4, C2, CP6, CP4, CP2, TP8, P6, P4, P2, O2, and the symmetrical left locations), discarding electrodes and their symmetric locations on the other hemisphere, for which data were available for less than 5 (/8) IVH infants.

### 2.10 Statistical analysis

In healthy preterm neonates, our syllables evoke a complex series of 4 peaks with latencies at 84, 244, 378 and 576 ms, whose amplitude decreases with the repetition of the syllables in the trial, a phenomenom known as habituation or repetition suppression, also reported in post-term babies (Dehaene-Lambertz and Dehaene, 1994) and adults (Woods and Elmasian, 1986). Our goal was to study whether infants with IVH, compared to healthy preterms, displayed a neural response to syllables with similar peaks, and habituation pattern. However, lesions in function of their size and localisation, may distord the evoked response on the scalp by altering the diffusion of the electrical field. Thus the voltage topography in different IVH infants might differ from the normal population even if syllables are correctly processed. Therefore, in addition to statistical test on the ERP peak amplitude and latencies (Student’s *t*-test on combined frontal electrodes [FP1, FPz,FP2]), we considered the whole set of electrodes and we performed two analyses, the first one to address the amplitude of the response and the second one the topography. First, to examine whether habituation was also present, we computed the global field power (or spatial standard deviation, i.e. across electrodes) of the voltage at each time-point in each neonate. If a brain source is activated, a negative and a positive pole are observed on the scalp, increasing the voltage difference between electrodes and thus the standard deviation across electrodes (i.e. GFP). Therefore the neural activity peaks, which correspond to GFP maxima, can be determined independently of the voltage topography (Brunet et al., 2011; Murray et al., 2008; Skrandies, 1990). GFP is sensitive to the amplitude of the electrical activity and we previously showed that its value around the peaks decreased with the repetition of the syllables in healthy preterms (Mahmoudzadeh et al., 2017). Therefore to evaluate whether syllables can elicit a neural auditory response in IVH neonates and whether this ERP is modulated by syllable repetition, we averaged the GFP values across a 40 ms time-window centered on the peaks previously determined in the healthy infants (i.e. 84, 244, 378 and 576 ms, (Mahmoudzadeh et al., 2017)) and entered these values in an ANOVA with peaks (2–3–4) and repetition (1–3) as within-subject factors and group (IVH *vs*. Healthy) as between-subjects factor. The effect of repetition cannot be studied for peak 1, as peak 4 of the previous syllable peaks at 576 ms and consequently still presents a high amplitude at the latency of P1 (84 ms), given our SOA of 600 ms (Fig. 2).

Second, we examined the degree of similarity of the topographies of the responses in the IVH and healthy groups: We used a spatial filtering procedure (Schurger et al., 2013) in which the voltage map in each subject is compared to a template at each time point in order to determine the distance between both maps. To serve as templates (or spatial filters), we created three 2D maps corresponding to the peaks 2, 3, and 4 in the healthy group: The voltage was averaged across a 40 ms time-window centered on each peak following S1 and normalized, to create three template maps. The template is performed from the healthy data. Then each infant, healthy and IVH, is compared to the template. In the case of the healthy infants, the template is computed on all infants minus the one who is compared. Thus for each subject (healthy and IVH), the spatial filtering procedure is equivalent and provides the similarity between each infant and the template. The procedure is done for each time-point over the entire trial for each map and not restricted to the time-window on which each template was computed, as we might have expected delayed responses in IVH infants.Note that, for each of the healthy neonates, the templates were computed by excluding the infant considered (i.e. on 18 infants instead of 19) to avoid data dependency. Finally, each of the three time-series was averaged in each infant over a 100 ms time-window centered on the peak value (244, 378 or 576 ms) after each of the four syllables, then entered in an ANOVA with repetition (1–4) as within-subject factor and group (IVH vs Healthy) as between-subject factor.

## 3 Results

### 3.1 Cerebral hemodynamic responses to syllables in preterms

As previously reported, the hemodynamic response in healthy preterm neonates consists of an increase in HbO and a decrease in Hb, which peak at around 7 s after onset of stimulation with specific patterns depending on the channel location over the right and left perisylvian areas (Mahmoudzadeh et al., 2013) for a detailed description of these results). A weak response was observed in IVH preterms (see Fig. 3B, video S1): AUCs (Fig. 3C) were significantly smaller in both hemispheres (LH *p <* 0.005, RH *p <* 0.01) during the first 10 s of stimulation in IVH preterms compared to healthy neonates. The response was also less extensive with significant activations only observed in the right Superior Temporal Gyrus (STG) in IVH infants contrasting with the widespread response recorded over all channels in healthy preterms (video S1, Fig. S1). We found no significant association between response magnitude and the location and extension of IVH. This is due, at least in part, to the weak hemodynamic response in IVH population making a statistical analysis of the magnitude difficult. Nevertheless, in IVH preterms, the RH variance was much greater than LH with an interquartile range (IQR) of (IQR_RH_ = 1.35 vs IQR_LH_ = 0.5; median_RH_ = 0.59 vs median_LH_ = 0.41) indicating greater variability of hemodynamic response in the right hemisphere than in the left hemisphere.

### 3.2 Event-related potentials

In healthy preterm neonates, four peaks were recorded at 84, 244, 378 and 576 ms after each syllable with a decrease in the amplitude of the peak when syllables were repeated (Mahmoudzadeh et al., 2017). Fig. 2B (right) shows that the ERP peaks and through have lower amplitudes and longer latencies in IVH group over the frontal electreodes (FP1- FPz-FP2). The global field power averaged around these peaks after each of the four syllables constituting a trial was compared in healthy and pathological infants (see Fig. 2D). No group effect was observed (F(1,25) <1). A main effect of repetition was observed (F(3,75) = 14.5, p < 0.001, which did not interact with the group (F(3,75) < 1). In both groups, the effect of repetition was significant (healthy F(3,54) = 10.04, p < 0.001 and IVH F(3,21) = 5.13, p = .008) demonstrating a similar decrease of voltage with syllable repetition (Fig. 2D). In healthy subjects, the amplitude decrease was significant by the first syllable repetition (F(1,18) = 16.22, p < 0.001) and was similar for the three peaks (peaks × repetition F(2,36) = 1.81, p = .18). In IVH infants, a main effect of peak (F(2,14) = 5.56, p = .017) and a significant peak × repetition interaction were observed (F(2,14) = 6.50, p = .01), related to a significant decrease of amplitude between the first and second syllables which was only observed for peak 4 F(1,7) = 9.8, p = .017). The same tendency was observed in healthy infants: when each peak was analyzed separately in this group, the most marked amplitude decrease was observed for peak 4 (F(1,18) = 11.27, p = .003, peak 3: F(1,18) = 3.14, p = .09, peak 2: F(1,18) = 2.59, p = .12).

To detect topographical differences between the two groups, we used a spatial filtering technique in which each subject’s response was compared to 2D maps of the peaks obtained from the healthy group grand average (see method, Fig. 2E). No significant group effect was observed for any of the filters (group effect for peak 2 spatial filter: F(1,25) <1; peak 3: F(1,25) <1; peak 4: F(1,25) = 1.17, p = .29).

## 4 Discussion

The results of this pilot study demonstrate a clear disconnection between neural and vascular responses in IVH neonates. While it was possible to record a neural response following each of the syllable, the hemodynamic response was almost completely nonexistent. As a group, IVH infants displayed sufficient synchronized activity after each syllable of the trials to obtain reproducible voltage maps, not statiscally different in topography and amplitude from those in healthy infants. This similarity does not preclude that IVH may impact neural responses and syllable perception. Indeed we observed a significant decrease of amplitude and an increase of latency over frontal electrodes in the IVH group relative to healthy group. This observation can be related to a genuine impairment of syllable processing in some, if not all, of IVH neonates but also to a distorsion of the surface voltage by the lesion or to the different background activity observed in IVH premature neonates, such as decreases in dynamic and increases in discontinuity and occurrence of abnormal EEG features (e.g. Positive Rolandic Sharp Waves (PRSW (Table 1 vs. 3: EEG is abnormal in 7 out of 8 preterm with IVH). The small number of subjects in our IVH group prevents to disentangle these different causes. The point we underline here is the discrepancy between neural and vascular responses: Whereas we were still recording a sizable neural response in these infants despite their high-grade IVH, the hemodynamic response was markedly reduced in terms of both amplitude and extent, pointing to a lack of functional neuro-vascular coupling. The absence of an exact physical model of preterm tissue conductivities (e.g., fontanel vs. skull) and brain gyrification, limited us from performing precise exogenous ERP source reconstructions. Although, uncertainty about electrical properties of head tissues (especially fontanel) is a challenging issue in the source localization in preemies (Azizollahi et al., 2016), previous NIRS and ERP studies (Mahmoudzadeh et al., 2013; Mahmoudzadeh et al., 2017) were consistent with the perisylvian lateralisation of structures involved in language discrimination (leftward for phonemes and rightward for voice), suggesting that similar generators are probed by both technics. The only area in which a neurovascular coupling was still detectable in IVH neonates was the right STG. The response to sounds is largely bilateral in healthy post-term infants. Asymmetries favouring the left, or right, hemisphere are limited to the most posterior region of the superior temporal lobe and in the planum temporale (Dehaene-Lambertz et al., 2010; Perani et al., 2010). Because the side and size of the asymmetry are dependent on the stimulus, they are hypothesized to be related to neural processing. By contrast, before term, hemispheric asymmetries appear to be more general and favouring the right hemisphere leading to the hypothesis of a faster maturation of this hemisphere during this period. Indeed, sulci generally appear one or two weeks earlier in the right hemisphere than in the left (Chi et al., 1977; Dubois et al., 2009). Microstructural changes related to grey matter maturation, captured with structural MRI reveal a faster maturation of the right relative to left in Heschl’ gyrus and STS during the first weeks of life. Cerebral blood flow is also larger (Lin et al., 2016) and the EEG power higher in the right hemisphere relative to the left before term. Finally, in a previous paper we reported larger amplitude of the hemodynamic response to syllables using the same paradigm in all right regions relative to left excepts the posterior temporal region, in which the left response was faster and more prolonged than the right but of similar amplitude (see Fig. 2 in (Mahmoudzadeh et al., 2013)). Thus, this persistent right STG response might be related to a more mature right hemisphere at this age (Ardila and Ostrosky-Solis, 1984) whose vascular network might have been more developed and thus resistant at the time of the initial hypoxic event. Abnormal neurovascular coupling to pathological spontaneous EEG discontinuity has already been reported in IVH premature neonates (Roche-Labarbe et al., 2007). Here, we observed that there is also an inability of the IVH brain to modulate its vascular supply in response to an external gentle stimulation, i.e. series of brief syllables presented at normal hearing level.

Brain oxygen saturation at rest was not different from that observed in controls (Fig. 3A), suggesting that cerebral hypoxia was probably not responsible for the altered cerebral hemodynamics. More likely the absence of hemodynamic responses to syllabic stimuli might be due to an impaired fine tuning of the coupling between the neuronal and vascular compartments (Roche-Labarbe et al., 2007), or an altered vascular function (e.g. elasticity) or an impairement of the cerebral autoregulation in IVH preterms (Perlman et al., 1983; Van Bel et al., 1987). At rest, many studies from different imaging modalities have described altered vascular function consisting in a decrease in CBF and CBV in IVH preterms: ^133^Xe clearance (Ment et al., 1981; Pryds et al., 1989), positron emission tomography (PET) (Volpe et al., 1983), echocardiographic measurement (Osborn et al., 2003) and fNIRS (Meek et al., 1999). Recently decrease in CMRO2 was also observed concomitantly with a decrease in CBF using combined DCS-NIRS measurements (Lin et al., 2016). This lack of oxygen compensation needed by the neural tissue might result in a decrease in neuronal activity at rest and even in cellular death (including astrocytes) due to hemorrhage (Gram et al., 2014). Such impacy on neuronal activity is well known in IVH patients, notably grade III and IV in whom disorganized EEG with pathological discontinuities are observed (Olischar et al., 2007; Tich et al., 2007). In preterm infants, most neurons have not achieved their final destinations and, even when they have reached their target location, their functional maturation and connectivity have not yet been completed. Similarly, astrocytes are also immature, affecting their functional efficiency in terms of neurotransmitter reuptake and neurovascular coupling. Too many factors are currently unknown to propose a precise model of the neuronal and hemodynamic response at this age, but we can at least assume that hemorrhage might attenuate the neuronal response to the exogenous stimulus (shortage of neurotransmitters, reduced integrity of white and grey matter and functional network change metabolic supplement) leading to less numerous and less efficient neurons responding to the repeated stimulation as expressed by the changes in ERP amplitude and increased latencies in case of IVH. Unfortunately because of ethical issues in these fragile premature, MRI was not feasible to evaluate mesoscopically the neuronal loss. Thus the reference image tool rely on the ultrasounds that have poor spatial resolution. In the context of our paradigm, repeated syllables increase the neural activity even to a lesser extent, the resulting increased metabolic demand should therefore had increased (even to a lesser extent) the amplitude of the hemodynamic response whatever the amount of neuronal loss. It is accepted that the quantitative coupling relationship is largely influenced by the type and dosage of anesthesia, including the actions on neural processing, vasoactive signal transmission, and vascular reactivity (Masamoto and Kanno, 2012). Therefore an effect of anesthetic cannot be completely ruled out. Only 3 out of 9 IVH premature were under sedation (Table 2) which is not different than in the control population. But because ERPs are only scantly modified compared to hemodynamic responses, it would suggest that, if it has any effect, the sedation may affect more the circulatory system than the neural auditory one (Table 3).

**Table 2:** Additional clinical features of the IVH infants tested.

Infant No.	Ultrasound Time before exam^a^	Evidence of progression of GMH-IVH on repeated head US	Blood Glucose (mmol/L)	Blood Gas (mmHg)	Blood pH	Hemoglobin level (g/dl)	Ventilatory support and/or mechanical ventilation^b^	Treatments with possible interfering effect	seizures and/or AED treatments
				pCO2					
				pO_2_					
1	D-1: IVH3	D + 8: IVH3	4.2	36.1	7.27	10.6	IMV	celestene	–
		D + 14: IVH3		123					
2	D-0: IVH3	D + 5: IVH4	4.7	48.3	7.16	7.7	CPAP	celestene	–
				64					
3	D-9: IVH3	D + 6: Tetra ventricular hydrocephaly and bilateral IVH	7.8	38.6	7.27	9	IMV	celestene	–
				62.2					
4	D-9: IVH3	D + 0: asymmetry of lateral ventricle (L > R) D + 8: IVH 3	4.8	52.8	7.32	8	IMV	celestene hypnovel,	–
				39.1				sufentanyl	
5	D-3: IVH4	D + 5: IVH4	2.6	66.8	7.28	13.2	AA	celestene	–
				49.2					
6	D-5: IVH4	D + 3: IVH4	5.3	37	7.34	11.6	IMV	celestene	–
				93.7				sufentanyl	
7	D-17: IVH3	D + 8: IVH4	4.6	33.3	7.28	12.4	IMV	celestene	–
				128					
8	D-2: IVH3	D + 5: IVH3	4.9	54.2	7.28	12.4	IMV	hypnovel,	–
				107				sufentanyl	

a “D” is fNIRS exam Day (e.g. D-1: one day prior exam).

b CPAP: Continuous positive airway pressure, IMV: Intermittent Mandatory Ventilation, AA: Ambient Air.

**Table 3:** Clinical features of the healthy infants tested.

Infant No.	Gender	GA at birth (weeks)	GA at test (weeks)	Birth Weight (g)	Apgar (1 min)	Apgar (5 min)	Brain US	Delivery	Presentation	Clinical conditions (Etiology)
1	M	32	32 2/7	1530	9	7	N	vaginal	transverse	twin
2	F	28 1/7	28 5/7	1490	9	10	N	cesarean	cephalic	RPH
3	M	32	32 2/7	1550	0	6	N	cesarean	cephalic	preeclampsia
4	M	32	32 3/7	2300	10	10	N	vaginal	cephalic	PROM
5	M	30 6/7	31 1/7	990	8	9	N	cesarean	cephalic	preeclampsia
6	F	31	31 4/7	1260	10	10	N	cesarean	cephalic	preeclampsia
7	F	31	31 2/7	1560	8	10	N	cesarean	breech	Anoxia-ischemia
8	M	29	29 6/7	1610	10	10	N	vaginal	cephalic	twin, preeclampsia, PROM
9	M	31 1/7	31 4/7	2300	10	10	N	vaginal	breech	twin
10	F	28	28 3/7	995	8	8	N	vaginal	cephalic	twin, preeclampsia
11	M	28	28 4/7	760	7	10	N	cesarean	cephalic	preeclampsia
12	M	30 2/7	30 5/7	1440	9	10	N	vaginal	cephalic	PROM, chorioamnionitis
13	M	32	32 1/7	1200	10	10	N	cesarean	cephalic	twin
14	M	30 2/7	30 5/7	1730	7	8	N	vaginal	cephalic	PROM
15	M	28 2/7	28 3/7	1340	8	8	N	cesarean	cephalic	twin
16	M	29 5/7	30 2/7	1560	7	8	N	vaginal	Cephalic	twin
17	F	30 3/7	30 6/7	1630	10	10	N	cesarean	Cephalic	twin
18	M	31 4/7	31 6/7	1090	10	10	N	cesarean	Cephalic	preeclampsia
19	F	31 1/7	31 4/7	2120	9	9	N	vaginal	Breech	PROM

M: Male, F: Female, GA: Gestational Age, EEG: ElectroEncephaloGram, Cranial US: Cranial ultrasound, N: Normal, RPH: RetroPlacental Hematoma, PROM: Premature Rupture Of Membranes.

Several studies have already shown the effects of IVH of preterm infants on oxygenation and cerebral hemodynamics. Lin et al. (Lin et al., 2016) have observed consistently lower CBF*_i_* and CMRO2*_i_* in ELGA neonates with low-grade GM-IVH compared to neonates without hemorrhages. Verhagen et al. (Verhagen et al., 2010) have shown that preterm infants with GMH-IVH had lower regional cerebral oxygenation (rcSO2) and higher fractional tissue oxygen extraction (FTOE) during the first 2 weeks after birth irrespective of the grade of GMH-IVH. This suggests that cerebral perfusion is decreased persistently for 2 weeks in infants with GMH-IVH, even in the presence of mild hemorrhages. The mismatch between neuronal and vascular responses suggests a different impact of cerebral hemodynamics instabilityon the neuronal and vascular systems. Whereas an cerebral hemodynamics instability may create a focal neural lesion, its effect on the vascular system might be more global by inhibibiting its capacities to adapt to neuronal activation, even at distance of the initial lesion. This general lack of modulation of the vascular supply may participate to the well-known neurodevelopmental difficulties of prematurely born infants, especially when they have experienced IVH (Marret et al., 2013). By decreasing or even disabling the neurovascular coupling in response to endogenous (Roche-Labarbe et al., 2007) or external sensory stimuli (as in the present study), IVH may induce further neuronal dysfunction in addition to that due to immaturity (Therien et al., 2004), and may constitute a supplementary risk factor for cerebral palsy, hearing impairment, lower IQ and lower vocabulary, reading and mathematical achievement test scores observed in prematurely born children (Marret et al., 2013).

## 5 Conclusion

This study demonstrates the value of optical imaging to identify cerebral hemodynamic abnormalities in critically ill preterms before behavioral changes are manifested or only minor abnormalities on other functional monitoring techniques such as EEG are visible. In situations of vascular disorders, such as grade I and II IVH, patent ductus arteriosus and ischemic stroke, EEG and ERP may be either normal or only transiently altered. In this context, multimodal imaging combining HD functional optical imaging and HD-ERP might be helpfull to more clearly understand the whole neurovascular unit (neuronal and vascular), its pathology and its deleterious impact. Larger multicentric studies are required to further and more precisely evaluate the effect of different level of IVH on the brain electrical and hemodynamic responses to different stimuli. As these infants are particularly fragile and difficult to move to a scanner, fNIRS might represent an alternative to fMRI (Wallois et al., 2012).

## Author contribution statement

F.W., G.D.-L. and M.M. designed research; M.M., G.K. and S.G. performed research; M.M., G.D.-L. and M.F. analyzed data; and M.M., G.D.-L., and F.W. wrote the paper.

## Disclosure/Conflict of interest

The authors have indicated they have no financial relationships relevant to this article to disclose. The other authors have no conflicts of interest to disclose.
