# Supplementary material for: Consequence of intraventricular hemorrhage on neurovascular coupling evoked by speech syllables in preterm neonates
Source: Dev Cogn Neurosci. 2018 Jan 5;30:60–9. doi: 10.1016/j.dcn.2018.01.001 (PMC6969131; doi:10.1016/j.dcn.2018.01.001)
Supplement: Supplementary file 1 [file mmc1.doc]

***Supplemental Information***

***
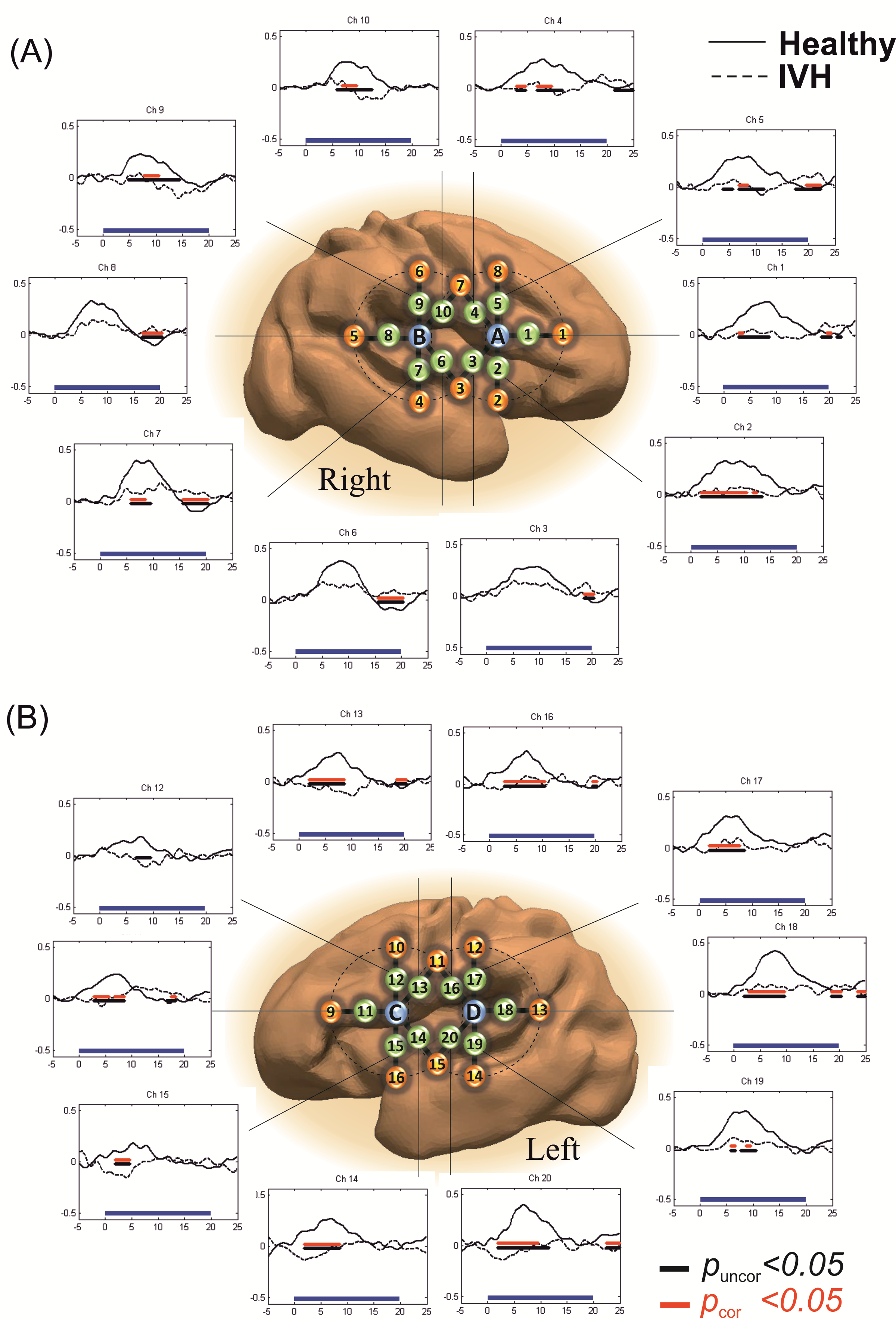
***

**Fig. S1.** Comparison of the hemodynamic responses to auditory stimuli in healthy vs. IVH premature infants. The time-courses of the grand average of HbO in healthy (solid line) and IVH (dotted line) infants are plotted for each channel. The y-axis represents the concentration changes in arbitrary units (a.u.). The x-axis displays the time in seconds. The blue rectangle along the x-axis indicates the duration of the stimulation block (0–20 s) and the black and red lines show significant clusters as determined by a cluster-based analysis. **(A)** RH, right hemisphere; **(B)** LH, left hemisphere.

**Video S1.** Hemodynamic responses in the two groups of infants (Healthy *vs.* IVH). The red rectangle indicates the time-window during which auditory stimuli were presented to the infants.

**Still Image (Video S1)**. Hemodynamic response of the infants in the two groups (Healthy vs. IVH).
